# Inspection of the lens thickness with preoperative biometric measurements prevents an erroneous interpretation of posterior capsule during FLACS

**DOI:** 10.1038/s41598-021-89209-0

**Published:** 2021-05-06

**Authors:** Mei Kurosawa, Hiroshi Horiguchi, Takuya Shiba, Tadashi Nakano

**Affiliations:** 1 Department of Ophthalmology, The Jikei University School of Medicine, Tokyo, Japan; 2 Roppongi Shiba Eye Clinic, Tokyo, Japan

**Keywords:** Diseases, Medical research

## Abstract

Optical opacity reduces quality of biometry images, making it potentially difficult to find the correct location for irradiation during femtosecond laser-assisted cataract surgery (FLACS). After experiencing a case of posterior capsule (PC) rupture because of optical opacity, we started lens thickness (LT) inspection, which indicates comparison of between intra- and pre-operatively measured LT. We retrospectively investigated the effectiveness of the LT inspection. One observer reviewed all FLACS treatment summaries for 3 years by CATALYS in the Jikei University Hospital, Tokyo. Based on the lines defining the PC on intraoperative OCT images, all cases were classified into three groups: undescribed, appropriate and inappropriate PC. Among the 1070 cases, 1047 cases had appropriate PC. In 19 cases, the PC line was undescribed because of dense cataract. Among 474 cases with no inspection, 4 cases had an inappropriate PC. Whereas, in 596 cases with the LT inspection, there was no case of an inappropriate PC. LT inspection significantly reduced the cases with inappropriate PC. The safety margins normally work to prevent severe complications. However, rare outlier cases had a high risk of severe complications. We propose LT inspection could be the most practical and convenient way for safety surgery.

## Introduction

Femtosecond laser-assisted cataract surgery (FLACS) takes advantage of capsulotomy and nuclear disassembly in phacoemulsification surgery. FLACS is considered to be easier and more reproducible than conventional cataract surgery^1^. However, some complications were reported particular in FLACS, such as capsule tag, anterior tear^2^, endothelial damage^3^ and capsular blockage syndrome during hydrodissection^4^, which were considered as being due to the uniqueness of FLACS.

One of the unique features of FLACS is an integration of the surgical laser with intraoperative biometry images^5^. The femtosecond laser platforms use imaging and software technologies to create a three-dimensional (3D) reconstruction of the cornea and the crystalline lens^6^. The area for laser-irradiation depends on the intraoperative biometry. The FLACS system needs an accurate 3D anatomical map from the cornea, anterior chamber, and lens to anterior vitreous to obtain a location for the femtosecond laser irradiation. To acquire high quality images, optical coherence tomography (OCT) is often used as the intraoperative biometry. We used CATALYS (Johnson & Johnson. New Brunswick, United States) as the femtosecond laser system and the intraoperative biometry was measured by spectral-domain OCT. To enhance the signal-to-noise ratio of the OCT images, the OCT sensors must receive photons as much as possible. Adequate mydriasis or long-duration scanning helps to obtain better OCT images. On the other hand, any opacity of the cornea, crystalline lens, and vitreous reduces the quality of the OCT images. For example, corneal opacity, which is edema, scarring, or folds, could diminish the quality of the image and cause the laser application to be incomplete^7,8^. Other optical opacity could also reduce OCT quality and make it difficult to find the correct location of the femtosecond laser irradiation. For nuclear disassembly during FLACS, it is considered that defining a location of the posterior capsule (PC) is quite important because the PC decides the depth of laser irradiation^5,7,9^. The PC is the deepest location in the anterior ocular segment and could be measured incorrectly due to any opacity from the cornea to the anterior vitreous. Since a high OCT intensity area in anterior vitreous looked like the PC and misled both an operator and the OCT subsystem of CATALYS to an erroneous interpretation, we experienced a case of PC rupture (PCR) caused by the direct femtosecond laser irradiation^10^ though the safety margins for an area of the femtosecond laser irradiation normally work to prevent severe complications even if the PC lines were inappropriately defined.

To prevent such severe complications, we have started to inspect the lens thickness before the femtosecond laser irradiation. We have been comparing intraoperatively acquired lens thickness during FLACS to the pre-operatively one acquired with other devices. To evaluate effectiveness of the lens thickness inspection for preventing erroneous interpretation, we reviewed all treatment summaries of femtosecond laser irradiation in our clinic. We found that the lens thickness inspection significantly reduced an erroneous interpretation of the PC and prevented severe complications based on this erroneous interpretation of the intraoperative biometrical measurements accordingly.

## Results

### The lens thickness inspection prevents an erroneous interpretation of a posterior capsule

FLACS was performed for 1070 eyes of 734 patients in this sequential study. Three hundred and seventy-seven were men and the mean age was 65.9 ± 12.7 (range 17.7–94.1). The average axial length of all patients was 24.85 ± 2.10 mm (range 21.28–34.03) as measured by IOL Master 700.

**Figure 1:**
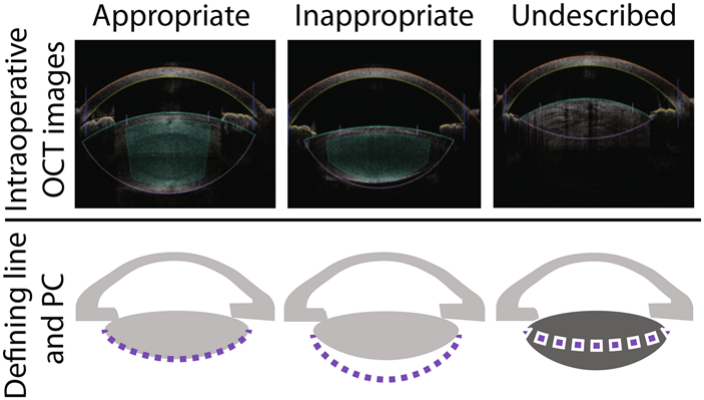
The lines defining the posterior capsule (PC) were classified into three groups by visual inspection. The intraoperative integrated optical coherence tomography (OCT) images from the all treatment summaries were classified into three groups; appropriate PC line, inappropriate PC line and undescribed. An appropriate PC line was defined as the line defining PC accurately aligned with the PC of the intraoperative OCT image (left column). An inappropriate PC line indicates the line defining PC clearly misaligned with the PC of the intraoperative OCT image (middle column). Because of dense cataract, the line defining the PC is automatically defined as minimum lens thickness (right column: undescribed).

**Figure 2:**
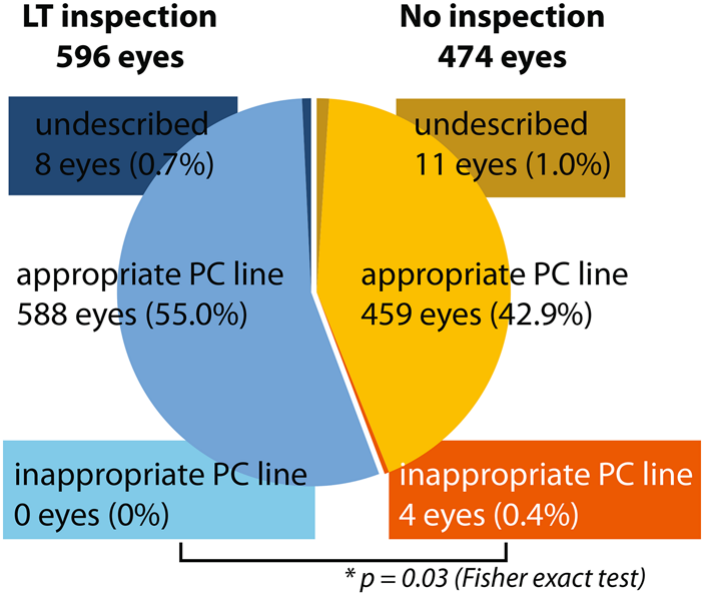
Overview results based on defining the PC line. All eyes are classified into two groups based on with or without the lens thickness inspection. The Right and the left half of the circle graph indicate the No inspection (warm colors) and the LT inspection group (cold colors), respectively. Using the CATARYS OCT subsystem, we are able to describe PC lines approximately 98%. Inspecting with the preoperative OCT image significantly reduces the number of cases that have a possibility to cause a severe complication (inappropriate PC line).

First, we classified all cases into three groups based on the relationship between the lines defining the PC and the intraoperative OCT images (Fig. 1). Among the total 1070 cases, 1047 cases (97.9%) had an appropriate PC line. In nineteen cases, the PC was not clearly visualized due to mature cataracts and they belonged to the undescribed group (1.8%). Second, we classified all eyes into two groups the day after PCR due to laser irradiation since we started the lens thickness inspection, which involves comparing the lens thickness acquired with other devices preoperatively to the intraoperative measurement (Fig. 2). The cases until PCR were classified as the No inspection group (474 eyes). The appropriate PC line was found in 459 (42.9%) cases and another 11 eyes (1.0%) were undescribed PC line because of mature cataracts. The remaining 4 cases had an inappropriate PC line (0.4%). The LT inspection group after the case of PCR had 596 eyes accordingly. Five hundred and eighty-eight (55.0%) of them had an appropriate PC line and the other 8 eyes had an undescribed PC line (0.7%). No one had an inappropriate PC line in the LT inspection group.

There was no significant difference in the rate of the undescribed PC line to the visualization between the No inspection and the LT inspection groups (11/463 eyes, 2.4% in the No inspection, 8/588 eyes, 1.4% in the LT inspection, respectively; p = 0.25, Fisher exact test). On the other hand, the rate of the inappropriate PC line in the No inspection group (4/459 eyes, 0.9%) was significantly higher than that in the LT inspection group (0/588 eye) (p = 0.03, Fisher exact test).

### Comparison of intra- and pre-operative biometric measurements for lens thickness

**Figure 3:**
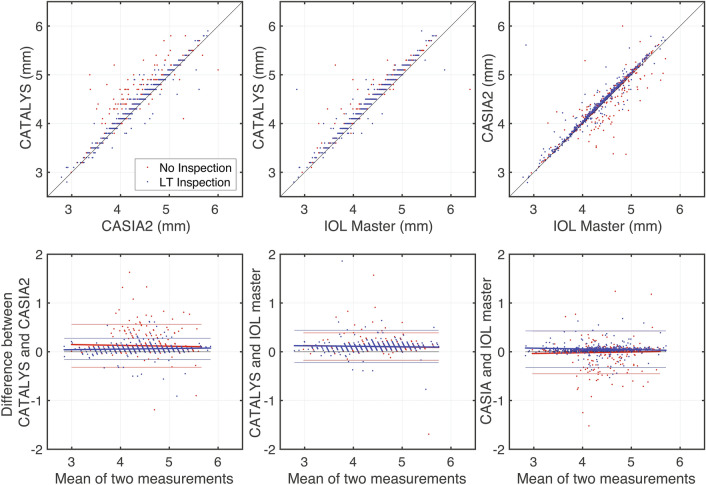
Comparison of the lens thickness measured by pre- and intra-operative OCT. The upper row of figures shows scatter plots comparing two measurements. The lens thickness by three different measurements are highly correlated. In the No inspection group (red dots), the lens thickness with CATALYS tends to be longer than those of CASIA2. However, the LT inspection reduces the differences between the lens thickness measured by CATALYS and CASIA2 (blue dots). The lower row of figures shows Bland—Altman plots. Red and blue dots indicate each measurement of the No inspection group and the LT inspection group, respectively. Most measurements exist between two of the thin solid lines, which indicate confidence intervals for 95% limits of agreement. Thick solid lines indicate the linear regression line. The LT inspection reduced the difference between the lens thicknesses as measured using CATALYS and CASIA2 (lower left panel). The lens thickness as measured using CATALYS is greater than that measured using IOL master, regardless of LT inspection (lower middle panel).

Inspection of the lens thickness before femtosecond laser irradiation reduced the ratio of the inappropriate PC line. To evaluate the impact of LT inspection, we depicted scatter plots of the lens thickness measured by IOL Master, CASIA2, and CATALYS, respectively (Fig. 3). Although the lens thickness measured by each device was highly correlated, the lens thickness measured by CATALYS with the No inspection (red dots, Fig. 3A) was slightly dispersed (p = 0.90). The mean lens thickness acquired with IOL Master, CASIA2, and CATALYS was 4.39 ± 0.54 mm (range 1.71–6.39), 4.42 ± 0.52 mm (range 2.79–6.11), and 4.48 ± 0.56 mm (range 2.5–5.9), respectively.

In the No inspection group, there was a significant difference among the lens thickness by these three biometric measurements (p < 0.001, Kruskal–Wallis test). The lens thickness measured by CATALYS was significantly higher than those measured by others (IOL Master versus CATALYS: p = 0.003, CASIA2 versus CATALYS: p = 0.002, Steel–Dwass test). In the LT inspection group, there was also a significant difference between the lens thicknesses measured by IOL Master (4.35 ± 0.53 mm) and CATALYS (4.46 ± 0.52 mm) (p = 0.0013, Steel–Dwass test). However, there was no significant difference between the lens thicknesses measured by CASIA2 (4.40 ± 0.52 mm) and CATALYS (p = 0.063). The mean lens thickness measured using CATALYS in the No inspection group was significantly higher than that in the LT inspection group (p = 0.0034, Wilcoxon rank-sum test).

To summarize, inspection of the lens thickness reduced the difference between the lens thicknesses as measured by CATALYS and CASIA2. The lens thickness measured by CATALYS was significantly higher than that measured by IOL master.

### Cases of inappropriate PC line and a possible case to avoid PCR

**Figure 4:**
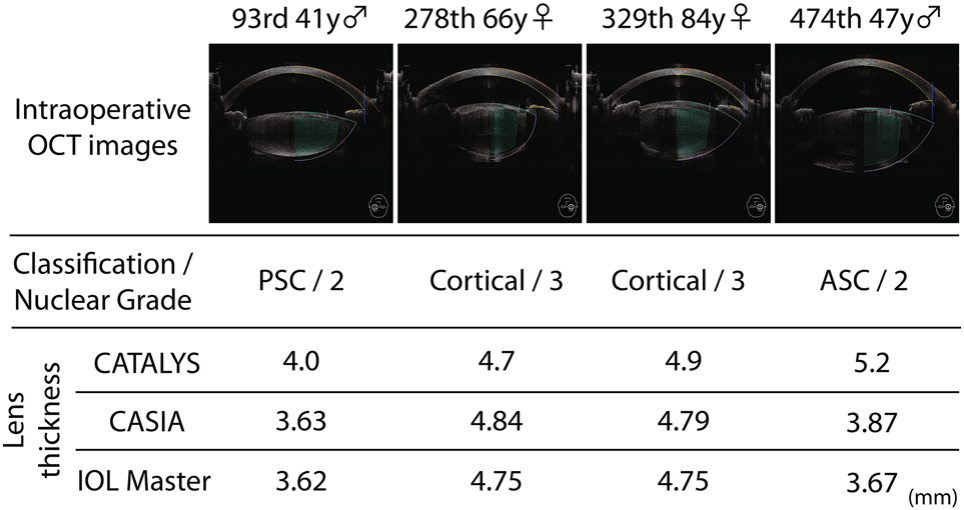
The details of cases with inappropriate PC lines. Four cases were classified as cases with inappropriate PC lines and their biometries were shown sequentially. In each of their intraoperative OCT images, the pure biometry images are shown on the left half of the image and the integrated images with the surgical laser area are shown on the right. In the second row, the classification of cataract and nuclear grade according to Emery-Little classification were described. Their lens thicknesses are shown in the third row. Except for the 278th case, three of them had longer lens thickness measured by CATALYS than other devices. The 93rd case and the 329th case are saved by the safety margin settled as 500 μm but posterior capsule rupture due to laser irradiation were occurred on the 474th case. (PSC; posterior sub-capsular cataract. ASC; anterior sub-capsular cataract, Cortical; cortical cataract).

Four cases were classified as the inappropriate PC line in this study. All of them were performed without the inspection of lens thickness. In three of the four cases, the intraoperative lens thickness was higher than that of the preoperative lens thickness. Hence, all these three cases had a risk of PCR due to laser irradiation (Fig. 4). Fortunately, the posterior capsular safety margin prevented such severe complications from occurring in the three cases; thus we did not notice any high-risk situations. However, the 474th case had eventually the complication of PCR due to laser irradiation^10^. After experiencing the complication, we started comparing lens thicknesses between intra- and pre-operative measurements before femtosecond laser irradiation. We encountered a possible case to avoid the complication of PCR by LT inspection (the 1029th case). In this case, the high OCT intensity area behind the PC misled the femtosecond laser system into defining the inappropriate PC line at first. Because of the lens thickness comparison, the surgeon immediately noticed the inappropriate PC line and manually adjusted the PC line to correct the segment on the OCT image (Supplementary Video S1).

## Discussion

Based on our retrospective assessment of the treatment summary, the ratio of the appropriate PC line was approximately 98% by CATALYS femtosecond laser system and surgeons in our clinic. Dense cataract prevented the PC line from being described in 1.7% of cases and definition of inappropriate PC line occurred in less than 0.5% of cases. After the occurrence of the severe complication, we started the lens thickness (LT) inspection to compare intra- and pre-operative OCT measurement before laser irradiation. The LT inspection reduced the ratio of cases with inappropriate PC lines and increased the correlation of lens thickness between pre- and intraoperative measurements presumably because the comparison helped our operators’ performance to detect the correct location of the PC.

In our study, three of the four cases with an inappropriate PC line had longer lens thickness measured by CATALYS than other devices. Usually a safety margin of the FLACS system could work for precaution to the direct laser irradiation to tissues that should not be irradiated. The safety margins are taken into account to prevent inadvertent injury to the cornea, iris, or posterior lens capsule^5^. Safety margins from the PC are typically 500–800 μm and automatically applied by the imaging platform and visualized on the OCT guidance for approval by surgeons before the laser is applied^7^. We predefined 500 μm as safety margins in our hospital. The safety margins saved the posterior capsule of two of three cases with inappropriate PC line from laser irradiation. However, the severe complication occurred in another case with an inappropriate PC line (474th case) due to direct laser irradiation to PC. The safety margins did not work for such cases with more than 1000 µm difference between inappropriate and correct PC lines, which indicates that other methods are required for safer operations in addition to safety margins. Another method for the safety operations might be a simple judgment of the lens thickness with intraoperative measurements during FLACS. For example, a case with longer lens thickness than some criteria would not be allowed for the irradiation of the femtosecond laser. We consider the method still has a possibility for complications because of several reports about lens thickness. Fukuda et al. reported lens thickness with cataract in Japanese measured by CASIA2 was 4.56 ± 0.39 mm^11^ and Haddad et al. showed lens thickness measured by CATALYS was 4.72 ± 0.41 mm (range 3.1–5.8 mm) at their hospital in the United States^12^. In our study, the range of the lens thickness varied from 2.5 to 5.9 mm with measurements by CATALYS, which is consistent with previous reports. The lens thickness of the case with the complication of PCR was 5.2 mm with CATALYS and within the standard range of the lens thickness. Hence, the simple judgment by the lens thickness measured with intraoperative OCT may not prevent severe complications. Here, we would propose one of the easy and simple ways to improve safety during FLACS. The LT inspection, which is just a comparison between intra- and pre-operatively measured lens thickness before the femtosecond laser irradiation. Though the LT inspection was not a complex way, the method significantly reduced both the rate of an inappropriate PC line and the risk of intraoperative complications during FLACS. We speculated that a reason to reduce the risk of complications is that surgeons easily noticed the correct anatomic structure once they received the caution by the LT inspection as shown in the movie appendix. In future, an artificial intelligence system could be developed to automatically confirm the real shape of the crystalline lens. We believe the LT inspection we introduced here was one of the practical and realistic methods currently.

Multiple reports have documented a learning curve when incorporating femtosecond laser cataract technology into a practice^1^. Our study included all the treatment summaries when we started to use the femtosecond laser system and the learning curve period was taken part in. The operator of PCR case was a highly experienced cataract surgeon (~ 10,000 conventional surgeries) and he had already performed over 100 cases of FLACS before the accident of PCR, which indicates that the surgeon had enough skill and knowledge for femtosecond laser systems. We believe that the complication of PCR due to direct laser irradiation could occur independently of a learning curve. Furthermore, we paradoxically speculated that the severe complication might have occurred because the surgeon had a lot of experiences with FLACS. This surgeon was so familiar with femtosecond laser systems which had high qualities of defining anterior segment with safety margins that he irradiated the laser as sequence flow without paying any strong attention to the shape of the posterior capsule and the lens thickness. If the case of PCR underwent surgery during the initial case for using the femtosecond laser system, he might be more careful of the segmentation and could avoid the severe complications. Hence, we would warn that such complication might increase with increasing the number of FLACS surgery without the LT inspection.

In this study, we used three unique optical methods to measure lens thickness. The three measurements correlated to but still slightly different from each other. We made the following speculations concerning the reason for a such difference among the three measurements. First, the optical biometry relies on partial coherence interferometry (PCI) in IOL master 700, swept source OCT (SS-OCT) in CASIA2, and spectral domain OCT in CATALYS. Second, there is no fixation point during measurements using CATALYS. Meanwhile, there is a fixation point during other measurements. Third, there are presumable differences in the software algorithms that define posterior capsule based on the acquired biometry images. Savini et al. reported that the mean value of the SS-OCT biometer was higher than that of the PCI^13^. Liao et al. also showed that the mean LT measured using SS-OCT was higher than that measured using PCI in the Bland–Altman analysis^14^. These results are consistent with our findings, which demonstrate that 4.35 and 4.40 mm in the LT inspection group are IOL master 700 (PCI) and CASIA2 (SS-OCT), respectively. Additionally, the mean LT measured using CATALYS in the No inspection group was significantly higher than that in LT inspection group. The LT inspection helped the operators determine the correct posterior capsule in some cases and reduced the mean of the LT measured using CATALYS. Consequently, the mean LT measured using CATALYS was not significantly different from the mean LT measured using CASIS2 but was still significantly higher than that measured using IOL master in our results.

Limitation of this study was the retrospective visualized inspection on treatment summary by one person. The visual inspection of the image depends on the insights of the observer. In future, an automatic judgment would be necessary independent from the detecting ability of an individual for the true posterior capsule from the biological intraoperative images. However, developing such algorithms could solve the issue of an erroneous interpretation of the tissue location simultaneously.

## Methods

### Subjects

This retrospective sequential study analyzed treatment summaries of femtosecond laser assisted cataract surgery (FLACS) at the Department of Ophthalmology in Jikei University hospital. Written informed consent according to the Declaration of Helsinki was obtained from each patient. We used CATALYS (Johnson & Johnson. New Brunswick, United States) as a femtosecond laser system. The CATALYS femtosecond laser system automatically saves treatment summaries after completion of all performances. The treatment summary describes the treatment details such as the day of birth, treatment day, the acquired OCT images and the integration of the surgical laser location with the intraoperative biometry. We reviewed all treatment summaries from June 2016 to March 2019. FLACS was performed by 8 highly experienced cataract surgeons (H.T, A.W, T.S, Y.M, T.O, Y.I, T.W, and M.K), who received training and received permission to use the device.

This study was approved by the institutional review board of the Jikei University School of Medicine (approval number: 27–331(8216)). The inclusion criteria for femtosecond laser-assisted cataract surgery at our hospital were (1) presence of cataracts and (2) written informed consent provided. The exclusion criteria were (1) small pupil dilation of < 4.5 mm, (2) significant corneal opacity, (3) small palpebral fissure, (4) noncooperation, or (5) any other conditions that warranted exclusion based on the operator's judgement.

### Biometric measurements

Preoperative biometry measurements as axial length (AL) and lens thickness were collected in all the cases. We routinely measured the lens thickness using IOL Master 700 (Carl Zeiss Meditec AG. Jena, Germany) and anterior swept source OCT imaging (CASIA2, TOMEY, Japan) and the AL using IOL Master 700.

The intraoperative OCT subsystem of CATALYS employs an 820–930-nm spectral domain OCT that takes 7 s to scan the anterior ocular segment accordingly. The device for docking an eye to the laser system is called a patient interface and it helps in preventing eye movement. The design of a patient interface is noncontact (non-applanation) in CATALYS. Patients are required to lie flat on the table with minimal neck support for proper docking and acquisition of a better resolution of intraoperative OCT images. The acquired OCT images are checked by the imaging system for proper refractive alignment and safety for correct identification^7^. The surgeon carefully looks at the monitor during the femtosecond laser systems obtaining intraoperative biometry and confirms the location of irradiation with acquired OCT images accordingly. The laser system automatically adjusts the segment lines after scanning the 3D OCT image of the anterior segment of the eye. The acquired intraoperative OCT images are then integrated with the surgical laser plan. In the integrated images, each segment is marked their own color: for example, a PC line and an area of lens fragmentation are described as the purple line and the green filled box, respectively. In addition, the safety margins are taken into account to save anterior tissues around laser irradiation from inadvertent injury^5^. We predefined 500 μm as the safety margins in our hospital. The surgeon, if needed manually adjusts each segment line on the OCT image visually after the automatic adjustments of the femtosecond laser systems. On adjusting the segment lines, the intraoperative OCT subsystem displays the lens thickness immediately based on the distance between the lines defining the PC and the anterior capsule respectively.

We had experienced a severe complication at the 474th case during our study. A posterior capsule rupture (PCR) occurred by the direct femtosecond laser irradiation because the line defining the PC misled both an operator and the OCT subsystem of CATALYS to erroneous interpretation^10^. Post this case of PCR due to laser irradiation, we started comparing the lens thickness both as intra- and pre-operatively measured with other devices before the femtosecond laser irradiation.

### Femtosecond laser instrument parameters

For anterior capsulotomy, the laser settings of the representative surgeon were: horizontal spot spacing, 5 μm; vertical spot spacing, 10 μm; and 4.0 μJ pulse energy. For lens fragmentation, the laser settings were: posterior capsule safety margin, 500 μm; anterior capsule safety margin 500 μm; horizontal spot spacing, 10 μm; vertical spot spacing, 40 μm; anterior pulse energy, 8 μJ; and 10 μJ posterior pulse energy.

### Phacoemulsification instrument parameters

The phacoemulsification machine parameters of the representative surgeon were as follows: the Whitestar Signature PRO (Johnson & Johnson Surgical Vision Inc.), which used a vacuum system, Venturi pump; vacuum pressure, 250 mmHg; irrigation pressure, 50 cm above eye level; an ultrasound power of zero; and a 21 G diameter curved tip.

### Analysis

We reviewed all treatment summaries that described treatment details of the acquired intraoperative OCT images, the lines defining each anterior segment of the eye, and the location of the femtosecond laser irradiation. One observer carefully investigated the lines defining the posterior capsule (PC) on the intraoperative integrated OCT images visually from the treatment summaries and classified all cases into three groups; appropriate PC line, inappropriate PC line and undescribed (Fig. 1). We defined an appropriate PC line as the line defining PC that almost accords with the PC of the intraoperative OCT image. On the other hand, an inappropriate PC line indicated that the line defining PC clearly misaligned with the PC of the intraoperative OCT images. When the visualization of the PC was prevented and the line defining PC was not correctly defined due to mature cataract which includes dense nuclear sclerotic cataracts and white cataract, we classified it as the undescribed group. If the imaging system was not able to detect the posterior lens surface and the lens thickness of the patient was not known, it would default to a conservative lens thickness value of 2.5 mm^12^. Hence, we excluded the undescribed group for the scatter plots of lens thickness accordingly.

Additionally, we classified all cases into two groups based on the operation day, which was the case of PCR due to laser irradiation (the 474th case) since we started the lens thickness inspection then. We compared intra- and pre-operatively measured lens thickness with other devices before the femtosecond laser irradiation. We named the latter as the ‘Lens thickness (LT) inspection’ group and the former as the ‘No inspection’ group respectively. Because both these groups could also be classified further into three groups based on the relationship between the line defining the PC and the intraoperative OCT image, and consequently, all cases belonged to one of the six groups.

Comparisons between the No inspection and the LT inspection groups with respect to the visualization of the PC line and the misalignment of the lines defining the PC on the intraoperative OCT image were performed by a Fisher exact test for 2 × 2 contingency tables. A Wilcoxon rank sum test was used to determine difference in lens thickness between the No inspection and the LT inspection groups. A Kruskal–Wallis test was used to determine differences in lens thickness between IOL Master, CASIA2, and CATALYS. A Steel–Dwass test was used for ad hoc multiple comparisons to compare biological data of lens thickness of IOL Master, CASIA2, and CATALYS before and after the case of PCR. The mean values (± standard deviation) of all measurements were calculated. A p value less than 0.05 was considered statistically significant.

## Supplementary Information

Supplementary Video S1.
